# Additional Physical Interventions to Conventional Physical Therapy in Parkinson’s Disease: A Systematic Review and Meta-Analysis of Randomized Clinical Trials

**DOI:** 10.3390/jcm9041038

**Published:** 2020-04-07

**Authors:** Ruben D. Hidalgo-Agudo, David Lucena-Anton, Carlos Luque-Moreno, Alberto Marcos Heredia-Rizo, Jose A. Moral-Munoz

**Affiliations:** 1 Department of Nursing and Physiotherapy, University of Cadiz, 11009 Cadiz, Spain; ruben.hidalgoagudo@alum.uca.es (R.D.H.-A.); carlos.luque@uca.es (C.L.-M.); joseantonio.moral@uca.es (J.A.M.-M.); 2 Department of Physiotherapy, Faculty of Nursing, Physiotherapy and Podiatry, University of Seville, 41009 Seville, Spain; amheredia@us.es; 3 Institute of Research and Innovation in Biomedical Sciences of the Province of Cadiz (INiBICA), University of Cadiz, 11009 Cadiz, Spain

**Keywords:** Parkinson’s Disease, Movement disorders, Physical therapy, Dance therapy, Aquatic therapy, Meta-analysis, Older adults

## Abstract

Parkinson’s disease (PD) represents the second most common neurodegenerative disease. Currently, conventional physical therapy is complemented by additional physical interventions with recreational components, improving different motor conditions in people with PD. This review aims to evaluate the effectiveness of additional physical interventions to conventional physical therapy in Parkinson’s disease. A systematic review and meta-analysis of randomized controlled trials were performed. The literature search was conducted in PubMed, Physiotherapy Evidence Database (PEDro), Scopus, SciELO and Web of Science. The PEDro scale was used to evaluate the methodological quality of the studies. A total of 11 randomized controlled trials were included in this review. Five of them contributed information to the meta-analysis. The statistical analysis showed favorable results for dance-based therapy in motor balance: (Timed Up and Go: standardized mean difference (SMD) = −1.16; 95% Confidence Interval (CI):(−2.30 to −0.03); Berg Balance Scale: SMD = 4.05; 95%CI:(1.34 to 6.75)). Aquatic interventions showed favorable results in balance confidence (Activities-Specific Balance Confidence: SMD=10.10; 95%CI:(2.27 to 17.93)). The results obtained in this review highlight the potential benefit of dance-based therapy in functional balance for people with Parkinson’s disease, recommending its incorporation in clinical practice. Nonetheless, many aspects require clarification through further research and high-quality studies on this subject.

## 1. Introduction

Parkinson’s disease (PD) is one of the major neurodegenerative disorders in older adults [1] and is the second most common neurodegenerative disease. PD usually appears at the approximate age of 55 and affects around 2–3/100 people over 65 years [2]. Moreover, the prevalence of PD could double by 2030 [3].

The main symptoms of PD are: resting tremor (regular and 4–8 cycles/second), extrapyramidal rigidity or hypertonia, bradykinesia or akinesia, postural instability, freezing of gait, and others such as sialorrhea, amimia, depression, and cognitive impairment [4,5,6]. Other symptoms that may be observed include altered postural reflexes, impaired balance, cognitive and neuropsychiatric disorders, sleep, speech and swallowing disorders, sensory disorders, and autonomic disturbances [4,6]. Regarding postural instability, PD patients present disorders in postural fixation and body sway even with strategies used to recover balance, especially in advanced stages of the disease [7,8]. They tend to adopt a flexion posture of the head and trunk and are unable to make postural adjustments to maintain the limits of stability [7,9]. People with PD often present gait dysfunctions such as decreased automaticity, reduction of step and stride length, limited center of mass and increased time in the double support phase [10,11]. Furthermore, they adopt a conservative strategy to handle obstacle crossing and external perturbations. Such dysfunctions put this population at higher risk of falls compared to age-matched healthy subjects [12]. With the progression of the disease, deterioration in axial manifestations appears, including alteration of postural reflexes and freezing, hypophonia, dysarthria, and dysphagia. According to Hely et al. [13], in their 20-year follow-up multicenter study performed in Sydney, in which 30 patients survived out of a total of 136 patients, 87% of the surviving patients experienced falls and 81% had freezing of gait. These progressive alterations constitute one of the main problems in advanced PD since they often do not respond to dopaminergic treatment in the same way as in the initial stages of the disease [14]. 

The main scales used to assess the stage and severity of PD are as follows: a) Hoehn and Yahr Scale [15], which describes the progress of PD symptoms and levels of disability, rating it in five stages (I–V). A higher score means greater disability. b) Unified Parkinson’s Disease Rating Scale (UPDRS) [16], which focuses on the assessment of PD signs and symptoms, on a 0–199 point scale, where a higher score on the UPDRS represents greater disability. The assessment is divided into four parts: (i) intellectual function, mood, behavior, (ii) activities of daily living (ADL), (iii) motor examination, and (iv) motor complications; and c) Schwab and England’s Activities of Daily Living Scale [17], which is a self-rated scale to assess the functional independence describing ADL capability, rated on an 11-point scale, from 0% to 100% ranging from vegetative state to fully independent. Rehabilitation programs and pharmacological treatment are considered to be beneficial for improving motor deficits in patients with PD [5]. In addition, Crizzle et al. [18] suggest that physical training is beneficial for improving the performance of ADL in PD patients, especially in the early stages, requiring the implementation of specific exercises to respond to specific previously evaluated deficits. Kwakkel et al. [19] emphasize that the main objective of the physiotherapist is to maximize the functional capacity of the patient and minimize secondary complications, in addition to adapting future rehabilitation programs to the patient’s domestic environment. Boonstra et al. [20], focusing on the functional improvement of gait and balance, highlighted the need to design multi-factorial protocols in which physical therapy plays a key role in correcting axial mobility deficits and preventing falls in people with PD. Recently, Tomlinson et al. [21] also highlighted the efficacy of short-term physical therapy in the treatment of PD, as opposed to non-intervention, exposing the need to provide evidence regarding specific techniques. Furthermore, Bloem et al. [22] remarked on the strong interest of the scientific community in these non-pharmacological treatments. However, although there are many scientific publications on the benefits of conventional physical therapy (CPT), it is unclear which additional physical interventions have significant effects on motor recovery in PD patients. Therefore, the main aim of this study is to deliver an overview of the current situation and the effectiveness of additional physical interventions beyond CPT in PD. We also describe and compare the different additional physical interventions used to improve the diverse motor conditions in people with PD.

## 2. Material and Methods

### 2.1. Search Strategy

This review follows the Preferred Reporting Items for Systematic Reviews and Meta-Analyses (PRISMA) [23] guidelines for systematic reviews of randomized controlled clinical trials (RCTs). The literature search was conducted during October–December 2017 in the following electronic databases: Web of Science, PubMed, Scopus, Scielo and Physiotherapy Evidence Database (PEDro). Different descriptor terms combined with Boolean operators were employed, as shown in Table 1. The search was restricted to clinical trials published during the last five years (2013–2017). No language filters were applied.

### 2.2. Selection Criteria

First, the PICO format was used to establish the selection criteria: (1) Population: adults with PD; (2) Intervention: physical interventions additional to CPT; (3) Comparison: group performing CPT according to the interventions included in the World Confederation for Physical Therapy statement [24], including therapeutic exercise (stretching, strengthening, proprioceptive, balance and walking training) and/or manual therapy techniques, such as joint mobilization or neuromuscular techniques; (4) Outcome: variables included in the different dimensions and domains of the International Classification of Functioning, Disability and Health (ICF) [25], specifically those related to body functions (static and dynamic balance, motor function) and activities and participation (mobility and gait, perception of falls, ADL, quality of life). All the included articles were RCTs. Articles were not considered for this review if: (1) participants with and without PD were analyzed together, but the outcome data were not available for participants with PD; (2) they included multidisciplinary interventions in which the specific impact of the physical intervention could not be extracted.

### 2.3. Study Selection Process and Data Extraction

First of all, the search was carried out by combining keywords in the databases mentioned above. Subsequently, duplicated articles were excluded. Titles and abstracts were then reviewed, and articles that did not meet the proposed selection criteria were excluded. The remaining articles were evaluated in more detail, and any that did not meet the criteria were excluded. Two reviewers (R.D.H.-A. and J.A.M.-M.) participated actively and independently in the process of study selection, review and systematic data extraction. Any disagreement was resolved by an additional reviewer (D.L.-A.).

The following data were extracted: (1) author and date of publication; (2) stage of the disease evaluated by the Hoehn & Yahr scale; (3) medical treatment; (4) number of participants included in the study groups; (5) characteristics of the interventions (type, frequency, duration of the session, duration of intervention, outcome measures, measuring instrument); and (6) the results obtained in each study.

### 2.4. Assessment of the Methodological Quality of the RCTs Included in the Review

The methodological quality assessment was conducted using the PEDro [26] scale. This has 11 items and each category is scored with 1 point if it meets the requirements, except for criterion number 1. A higher score indicates higher methodological quality. Thus, a study with a PEDro score of 6 or higher is considered to have a high level of quality (6–8: good; 9–10: excellent), and a study with a score of 5 or less is considered to have a low level of quality (4–5: acceptable; <4: poor) [27].

### 2.5. Statistical Analysis

The statistical software Review Manager (RevMan) 5.3 (The Cochrane Collaboration, The Nordic Cochrane Centre, London, United Kingdom) was employed for statistical analysis. Changes in the effect size between the intervention group and the comparison group (CPT) were analyzed in the meta-analysis. *p* < 0.05 was considered to be statistically significant. The standardized mean difference and the 95% confidence interval (CI) were calculated. The instructions of the Cochrane Handbook for Systematic Reviews of Interventions for obtaining standard deviation from confidence intervals and change-from-baseline standard deviation were employed when needed. A fixed-effect model was used when no heterogeneity was detected; a random model was used when homogeneity was determined. The chi-square test and the I^2^ statistic were used to determine the heterogeneity. Forest plots were used to represent the results of the meta-analyses. 

## 3. Results

**Figure 1:**
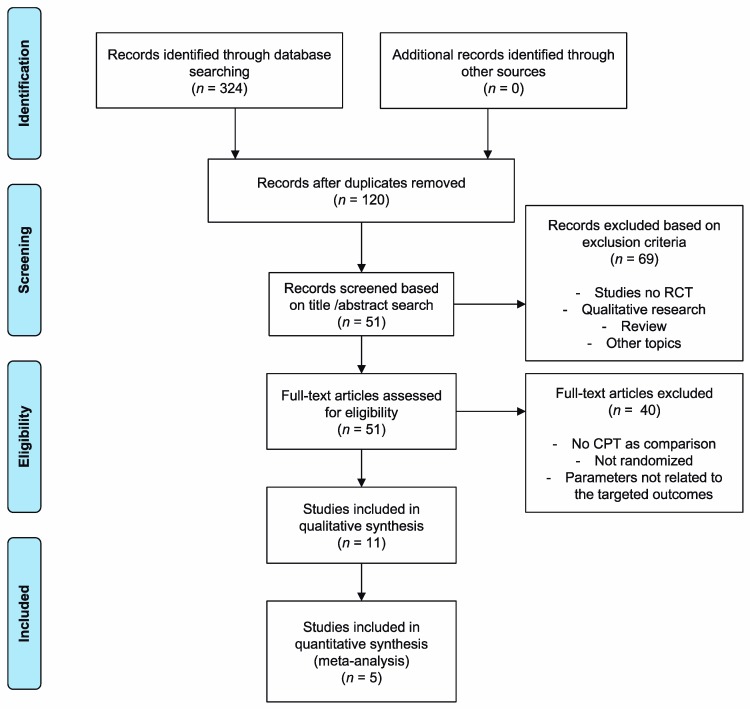
Flow diagram of the included studies.

Figure 1 illustrates the selection process of this systematic review and meta-analysis, retrieving a total of 324 potentially relevant articles.

### 3.1. Assessment of the Methodological Quality of the RCTs Included in the Review

The scores obtained on the PEDro scale are shown in Table 2. Nine RCTs had high methodological quality with scores on the scale equal to or greater than 6: Picelli et al. [11], Nimwegen et al. [28], Volpe et al. [29], Volpe et al. [30], Monticone et al. [31], Hashimoto et al. [32], Rios Romenets et al. [33], Volpe et al. [34], and Carpinella et al. [35]. Shujaat et al. [36] and Ricciardi et al. [10] scored 5, obtaining the lowest score.

### 3.2. RCT Inclusion and Classification

Table 3 presents the main characteristics of the study interventions. All included RCTs selected adult patients for research.

### 3.3. Groups Included in the Meta-Analysis

For statistical comparison, only those RCTs that measured the same variable with the same measuring instrument and also performed the same type of intervention in comparison with CPT were taken into account. Table 4 presents the six groups formed for meta-analysis, depending on the measurement instruments: UPDRS-III, Timed Up and Go (TUG), Berg Balance Scale (BBS), Activities-specific Balance Confidence Scale (ABC), Falls Efficacy Scale (FES), and Parkinson Disease Questionnaire (PDQ-39). These measurement instruments evaluate five different variables: motor function, balance, balance confidence, fall-related self-efficacy, and quality of life.

#### 3.3.1. Motor Function (UPDRS-III)

**Figure 2:**
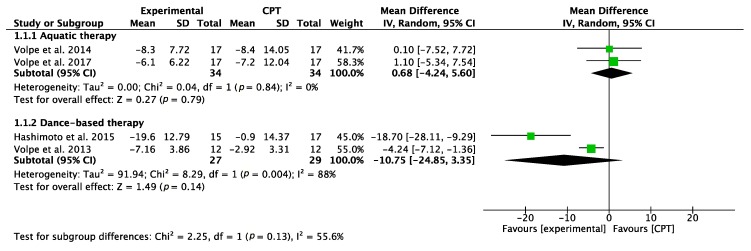
Forest plot for motor function measured by UPDRS-III. Green block indicates the weight assigned to the study and the horizontal line depicts the confidence interval. Black rhombus shows the overall result. The bold words highlight the total/subtotal values and the different modalities of therapies used in the interventions.

The effectiveness of different physical therapy interventions on motor function was analyzed using the UPDRS-III, as shown in Figure 2. Regarding the comparison of CPT with aquatic therapy (AT), the result of the meta-analysis was inconclusive. Concerning the effects of dance-based physical therapy (DT) on motor function, both Volpe et al. [29] and Hashimoto et al. [32] showed favorable results, with the second RCT presenting the best results. Nevertheless, the overall result of the meta-analysis was inconclusive.

#### 3.3.2. Balance (TUG and BBS)

Regarding the effects on walking balance and functional mobility analyzed using the TUG scale [38], a study by Volpe et al. [30] showed positive effects of AT on balance while a later study by the same authors [34] found no beneficial effects. The result of the meta-analysis was inconclusive. Concerning the DT interventions, Rios Romenets et al. [33] showed significant results when comparing the effects of DT intervention with those obtained through self-directed exercise. The meta-analysis revealed statistically significant results.

**Figure 3:**
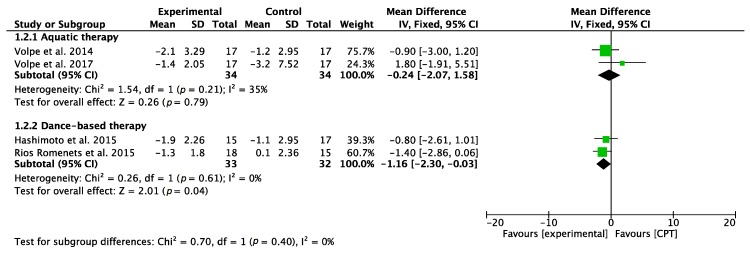
Forest plot for motor balance measured by TUG. Green block indicates the weight assigned to the study and the horizontal line depicts the confidence interval. Black rhombus shows the overall result. The bold words highlight the total/subtotal values and the different modalities of therapies used in the interventions.

**Figure 4:**
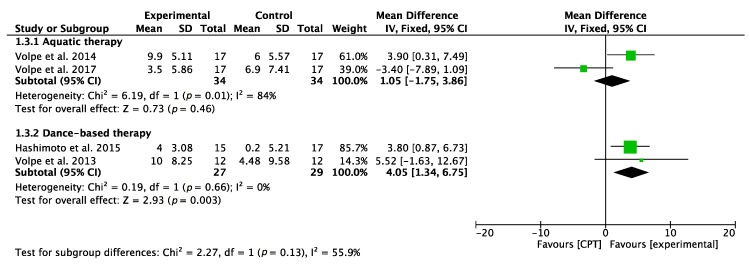
Forest plot for balance measured by BBS. Green block indicates the weight assigned to the study and the horizontal line depicts the confidence interval. Black rhombus shows the overall result. The bold words highlight the total/subtotal values and the different modalities of therapies used in the interventions.

Taking into account the interventions based on AT, similar results were obtained regarding the ability to maintain standing balance measured with BBS [39], the overall result of the meta-analysis being inconclusive. However, when analyzing DT interventions, both Volpe et al. [29] and Hashimoto et al. [32] showed significant results, with the second RCT showing the most significant results. The meta-analysis revealed statistically significant results. Figure 3 and Figure 4 show the results of the meta-analyses.

#### 3.3.3. Balance Confidence (ABC) 

**Figure 5:**
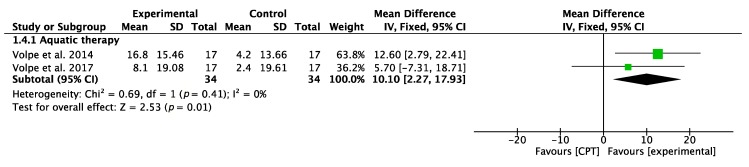
Forest plot for balance confidence measured by ABC. Green block indicates the weight assigned to the study and the horizontal line depicts the confidence interval. Black rhombus shows the overall result. The bold words highlight the total/subtotal values and the different modalities of therapies used in the interventions.

In this case, interventions based on AT gave favorable results after measurement with the ABC scale, the overall result of the meta-analysis being favorable, as shown in Figure 5. Both RCTs showed favorable results. 

#### 3.3.4. Fall-Related Self-Efficacy (FES)

**Figure 6:**
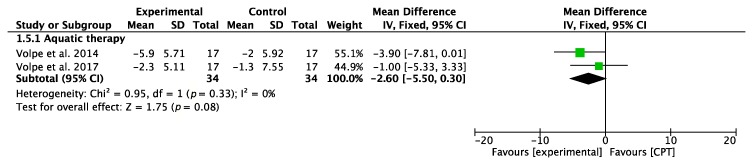
Forest plot for fall-related self-efficacy measured by FES. Green block indicates the weight assigned to the study and the horizontal line depicts the confidence interval. Black rhombus shows the overall result. The bold words highlight the total/subtotal values and the different modalities of therapies used in the interventions.

Concerning the fear of losing balance and suffering a fall, analyzed through the FES, the overall result of the meta-analysis was inconclusive, although Volpe et al. [30] reported significant results in favor of AT. The results of the meta-analysis are shown in Figure 6.

#### 3.3.5. Quality of Life (PDQ-39)

**Figure 7:**
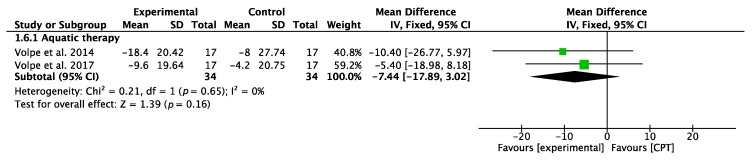
Forest plot for quality of life measured by PDQ-39. Green block indicates the weight assigned to the study and the horizontal line depicts the confidence interval. Black rhombus shows the overall result. The bold words highlight the total/subtotal values and the different modalities of therapies used in the interventions.

Finally, concerning improvements in quality of life, analyzed using the PDQ-39 questionnaire, AT interventions showed favorable results, but the overall result of the meta-analysis was inconclusive. Both RCTs showed favorable results. The results of the meta-analysis are shown in Figure 7.

## 4. Discussion

This paper aimed to synthesize the existing evidence on physical therapy in PD and to analyze the effect of additional therapies beyond CPT on different motor conditions. A total of 11 RCTs were reviewed, and these used different types of intervention, such as: AT, DT, physical therapy supported by the use of robotic assistance and virtual reality systems, treadmill interventions, and physical therapy supported by other disciplines such as occupational therapy or psychology. Due to the heterogeneity of parameters studied and the diversity of instruments and scales used in the assessment, only five RCTs were considered for statistical comparison through meta-analysis. The main findings of this study are discussed below.

Regarding the methodological quality of the studies, it should be highlighted that nine of the RCTs had good methodological quality. Moreover, all the RCTs included in the meta-analysis had a score of 6 or higher. PD patients could have heterogeneous characteristics depending on the levels of disability, the time since onset of disease, and the medical treatment administered. These factors could affect the results obtained in response to the physical interventions. Nevertheless, in our meta-analysis, all the RCTs [29,30,32,33,34] included patients that were scoring 1–3 on the Hoehn & Yahr Scale, and thus were in the early to mid-stage of PD, with mild to moderate disability, as well as patients that were stable as a result of drug therapy and were leading independent lives [14]. Concerning medical treatment, all included patients continued their usual medical treatment, which remained stable for the full study period, and they did not experience the side effects that appear during long-term treatment of dopaminergic medication (“on-off phenomenon”). In addition, to control factors that could affect the results, participants were excluded if their medication was modified during the study, and they were assessed at the same time each day. Thus, further trials based on physical interventions targeting PD patients in more advanced stages and those during the off-medication phase could increase the current evidence.

In terms of motor function, four RCTs analyzed the effects of different interventions in people with PD. In all cases, the system selected to compare results was the scale based on UPDRS-III motor parameters. The AT interventions seemed to be no more effective than CPT. However, interventions based on specific exercise routines from dance classes, as shown in the study by Hashimoto et al. [32], appeared to have greater effects on motor function than CPT. The CPT consisted of sessions based on coordinated joint mobility exercises, stretching, and gait re-education, among other basic protocols. It would be interesting to deepen our understanding of the neurological mechanisms involved in auditory feedback associated with the interpretation of the rhythmic patterns of dance that make this discipline an effective treatment in PD patients.

In terms of the effects of these interventions on balance, the main scales of measurement used were the BBS scale and the TUG. Once again, DT interventions had the best effects on balance, using both scales. Nevertheless, the results obtained using the TUG scale should be interpreted with caution as only Rios Romenets et al. [33] showed significant benefits. Dance seems to provide elements that positively condition the evolution of balance in patients with PD, although the sample sizes were still relatively small. Sample size should be increased in future research, and a reasonable follow-up will be required to verify the maintenance of long-term effects. The findings of this review coincide with those obtained by Shanahan et al. [40] and Aguiar et al. [41], which suggest that DT may be beneficial in improving mobility, balance, motor performance, and quality of life in people with PD, highlighting the importance of therapeutic adherence to these long-term treatments. In addition, both reviews refer to the need to explore the results based on the different disciplines or genres within dance. There is thus an opportunity to explore new and interesting lines of research to determine the beneficial type of exercise, duration, intensity, and frequency.

In contrast, interventions based on AT did not lead to conclusive improvements in balance, fall-related self-efficacy, and quality of life, although the results suggested some improvements with respect to CPT. Moreover, they led to improvements in balance confidence. These results should be interpreted with caution; the total sample size was too low to make firm recommendations. In this context, it should be noted that fear of falling could be a predictor of recurrent falls and a possible barrier to physical land-based exercises [42]. Methajarunon et al. [43] indicated specific benefits in parameters such as balance or gait, higher than those achieved in land-based physical therapy sessions. It would be interesting to delve deeper into the specific benefits of this therapy since interventions in this regard are very scarce.

Finally, it is apparent that high-quality studies are needed to determine the long-term effects of additional physical interventions beyond CPT. These interventions have shown some potential benefits, although the parameters are not well defined. They also have an important characteristic that might increase adherence to the therapeutic program, the ludic component. Thus, DT could be an enjoyable social activity that produces physical, emotional, and social benefits [44] since it simultaneously affects motor and cognitive function, as well as mental symptoms [32], factors that are strongly linked to quality of life in PD patients [45]. Furthermore, in line with Delabary et al. [46], the improvements discussed in this review regarding balance after DT highlight the possible benefits for functional mobility, independence, and functional autonomy in patients with PD. Future studies about DT should include in their programs specific and rhythmic activities, auditory and visual feedback, and different aspects to enhance the socialization of people with PD [46], and it would be interesting to analyze which kinds of dance genres are more effective for improving motor function, balance, and quality of life in PD patients.

Although the present study presents useful information, some limitations should be addressed. First, strict exclusion criteria were established, thus leaving out all studies that were not an RCT. Second, the heterogeneity of the included studies makes comparison difficult. For this reason, out of the total of 11 RCTs included, only five contributed information to the meta-analysis. This heterogeneity was present in both the intervention and control groups (the protocols of CPT differed), so the results should be interpreted with caution. In many cases, despite evaluating the same parameter, the difference in scales or instruments used makes it impossible to compare them statistically. Another relevant aspect to mention is the sample size, which was small in most studies.

## 5. Conclusions

There is no conclusive evidence on the effectiveness of AT interventions for improving motor function, balance, and quality of life in people with PD. However, improvements were obtained in subjective balance confidence.

Moreover, physical interventions based on DT seem to be more effective than those performed as CPT, especially for improving functional balance, so they should be incorporated into clinical practice. Further research is needed to elucidate which specific factors make DT an adequate intervention for improving motor-related disorders in adults with PD. In addition, further research with larger sample sizes and greater homogeneity in terms of the type of DT used, and the frequency and the duration of the interventions, is needed, as well as research to identify which specific factors of therapy have the greatest impact in achieving positive motor outcomes in adults with PD.

## Figures and Tables

**Table 1:** Search strategy.

Database	Records	Search Terms
PubMed	127	“Parkinson Disease”, “Physiotherapy” and “Physical Therapy”
PEDro	33	
WoS	108	
Scopus	31	
SciELO	25	

**Table 2:** Scores obtained after methodological evaluation according to the PEDro scale.

PEDro Scale												
Study	Total Score	1	2	3	4	5	6	7	8	9	10	11
Picelli et al. (2013) [11]	8	_	X	X	X			X	X	X	X	X
Nimwegen et al. (2013) [28]	8	_	X	X	X			X	X	X	X	X
Volpe et al. (2013) [29]	6	_	X	X	X			X			X	X
Shujaat et al. (2014) [36]	5	_	X		X				X		X	X
Volpe et al. (2014) [30]	8	_	X		X	X		X	X	X	X	X
Ricciardi et al. (2015) [10]	5	_	X		X			X			X	X
Monticone et al. (2015) [31]	6	_	X	X	X				X		X	X
Hashimoto et al. (2015) [32]	6	_	X	X	X			X			X	X
Rios Romenets et al. (2015) [33]	6	_	X		X				X	X	X	X
Volpe et al. (2017) [34]	6	_	X	X	X			X			X	X
Carpinella et al. (2017) [35]	6	_	X		X			X	X		X	X

The ‘X’ symbol indicates that the item met the criteria. Item 1 is related to external validity and not used in the method score.

**Table 3:** Main characteristics of the interventions.

Study	Stage *	Medical Treatment	Intervention **	Frequency	Session Duration	Intervention Duration	Outcome Measures	Measuring Instrument	Results
Nimwegen et al. (2013) [28]	1–3	Medical treatment was not described.	IG: *n* = 299 ParkFit program. CG: *n* = 287 CPT (Keus et al. (2007) [37] evidence-based recommendations)	7 days/week	ND	6, 12, 18, 24 months. months	Level of physical activity	LAPAQ questionnaire. PDQ-39, Daily Activity, Activity Monitor, Six-Minute Walk Test	There is no change in physical activity level after the ParkFit program. LAPAQ *p* = 0.19 (at 24 months). Secondary results suggest greater involvement in specific elements improving physical condition among the IG. Daily activity *p* < 0.001 Activity monitor *p* < 0.001 PDQ-39 *p* = 0.14 Six-minutes run *p* = 0.05
Picelli et al. (2013) [11]	3	Participants continued their usual medical treatment, which remained stable for the full study period. Patients with severe dyskinesias or “on-off” fluctuations, and changes of PD medication during the study, were excluded. Patients were tested and trained during the “on” phase, 1–2.5 h after taking their morning dose.	IG 1: *n* = 20 Robotic assistance. IG 2: *n* = 20. Treadmill training CG: *n* = 20. CPT (PNF concept focused on gait)	3 days/week	45 min	4 weeks	Ability to walk without assistance, travel speed, spatial-temporal gait, balance.	Main variables: 10MWT and 6MWT. Secondary variables: Spatial-temporal parameters for walking.	No significant evidence was found in the primary variables between the IG1 group and the IG2 group. Test 10MWT *p* = 0.869 Test 6MWT *p* = 0.941 Whether significant differences were found in the primary variables between the TGR and TT groups compared with PT. 10MWT: IG1 vs. CG *p* = 0.003 IG2 vs. CG *p* = 0.041 6MWT: IG1 vs. CG *p* = 0.021 IG2 vs. CG *p* = 0.048
Volpe et al. (2013) [29]	1–2.5	Participants continued their usual medical treatment, which remained stable for the full study period. Tests were performed at the same time of day.	IG: *n* = 12 Irish dance. CG: *n* = 12 CPT (KNGF Guidelines for physical therapy in PD)	1.5 h/week	90 min	6 months	Level of mobility, balance, quality of life.	UPDRS (engine), BBS, TUG, FOG, PDQ-39	Multicenter studies with larger sample sizes are needed to determine which therapy is most effective. UPDRS III (motor) *p* = 0.019 TUG test *p* = 0.007 BBS *p* = 0.051 FOG *p* = 0.000 PDQ-39 *p* = 0.153
Shujaat et al. (2014) [36]	1–3	Participants continued their usual medical treatment, which remained stable for the full study period.	IG: *n* = 24 Kayak intervention. CG: *n* = 24 CPT (strengthening exercise and core stability)	6 days/week	75 min	4 weeks	Ability to rotate cervical and thoracolumbar, mobility in bed.	Goniometer and MPAS	The rotation capacity at the cervical and thoracolumbar level is significantly increased in both groups after analyzing the measurements with the goniometer *p* < 0.001. Bed mobility is also significantly increased in both groups *p* < 0.001. The results are significantly better in the IG.
Volpe et al. (2014) [30]	2.5–3	Participants continued their usual medical treatment, which remained stable for the full study period. The evaluation of the different scales was performed one hour after the first dose of Levodopa (“on” medication phase).	IG: *n* = 17 Aquatic therapy CG: *n* = 17 CPT (KNGF Guidelines for physical therapy in PD)	5 days/week	60 min	2 months	Balance, functional capacity, motor capacity, number of falls, motor performance, ability to perform ADL.	Centre of pressure with stabilometric platform, UPDRS II and III, BBS, TUG(s), ABC, FES, PDQ-39, Falls diary.	Significant increases were observed in the variables analyzed in both groups *p* = <0.002. These improvements are significantly greater in the IG. Centre of pressure *p* = 0.05 BBS *p* = 0.005 ABC *p* = 0.0001 FES *p* = 0.003 PDQ-39 *p* = 0.006 Falls diary *p* = 0.001
Monticone et al. (2015) [31]	2.5–4	Participants continued their usual medical treatment, which remained stable for the full study period. Patients with previous “on-off” fluctuations were excluded. Patients were assessed during “on” state approximately 1 h after the first drug assumption.	IG: *n* = 32 Multidisciplinary rehabilitative care CG: *n* = 32 CPT (neuromotor techniques, joint mobilization, strengthening and stretching exercises, proprioceptive and walking training)	7 days/ week	IG and CG: 90 min. Physical training. IG: 30 min Psychology (2 sessions per week). 30 min occupational therapy 1 session per week	8 weeks	Motor development, balance, ability to perform ADL, quality of life.	MDS-UPDRS-III, BBS, FIM, PDQ-39	The multidisciplinary intervention shows better results in parameters such as motor development, balance, ADL and quality of life. MDS-UPDRS-III *p* < 0.001 BBS *p* < 0.001 FIM *p* < 0.001 PDQ-39 *p* < 0.012
Ricciardi et al. (2015) [10]	2–3	Participants continued their usual medical treatment, which remained stable for the full study period.	IG 1: *n* = 9 Best side improvement. IG 2: *n* = 9 Worst side improvement. CG: *n* = 10 CPT (strengthening and mobility exercises)	2 times/week	1 h	3 months	Motor development, balance, ability to perform ADL, quality of life.	UPDRS-III, Tinetti (total score), Tinetti (gait), GFQ, SPPB.	Better results are evidenced in IG1 UPDRS-III (IG1 vs CG) *p* = 0.01. Tinetti (total score) *p* = 0.05. Tinetti (gait) *p* = 0.01.
Hashimoto et al. (2015) [32]	IG and CG1: 2–3, CG2: 2–4.	Participants continued their usual medical treatment, which remained stable for the full study period. Participants whose medications changed during the study period were excluded from the analysis	IG: *n* = 15 Dance-based therapy. CG 1: *n* = 17 CPT (joint mobilization, balance and walking training by video or book) CG 2: *n* = 14 No intervention.	1 time/week	60 min	12 weeks	Motor and cognitive functions, mental symptoms related to Parkinson’s disease	TUG time, TUG step number, BBS, FAB, MRT response time, AS, SDS, UPDRS	Significant improvements are evident before and after the intervention in the IG dance group. TUG time *p* = 0.006 TUG step number *p* = 0.005 BBS *p* = 0.001 FAB *p* = 0.001 MRT response time *p* < 0.79 AS *p* < 0.001 SDS *p* = 0.006 UPDRS *p* < 0.001
Rios Romenets et al. (2015) [33]	1–3	Participants continued their usual medical treatment, which remained stable for the full study period.	IG: *n* = 18 Dance-based therapy. CG: *n* = 15 CPT (Pamphlet about PD exercise from the Parkinson Society of Canada)	IG. 2 times/week	IG. 1 h	12 weeks	General motor severity, other motor conditions, balance, cognitive level, fatigue, apathy, depression and quality of life.	MDS-UPDRS-III, General clinical impression by the patient, examiner evaluation, MiniBESTest, TUGtime, TUGstep, improvements in turns, MoCa, FES.	MDS-UPDRS-III *p* = 0.85 General clinical impression by patient *p* = 0.33 Examiner’s evaluation *p* = 0.02 MiniBESTest *p* = 0.032 TUGtime *p* = 0.042 TUGdts *p* = 0.012 Improvements in turns *p* = 0.066 MoCa *p* = 0.080 FES *p* = 0.057
Volpe et al. (2017) [34]	2–3	Participants continued their usual medical treatment, which remained stable for the full study period. Patients were always tested at the time of their optimal antiparkinsonian medication (‘on’ phase) and no change in medication was allowed during the study period.	IG: *n* = 15 Aquatic therapy. CG: *n* = 15 CPT (Postural realignment exercises)	5 times/week	60 min	8 weeks	Degrees of cervical and dorsal flexion, lateral inclination angle of the trunk. Motor symptoms, balance, balance confidence, fall-related self-efficacy, and quality of life.	Posturographic system and Body Analysis Kapture (BAK) System. UPDRS-III, BBS, ABC, TUG, FES, PDQ-39	No significant differences between groups. Statistical data supports significant improvements in IG: UPDRS-III *p* = 0.001 BBS *p* < 0.001 ABC *p* = 0.02 TUG *p* = 0.036 FES *p* = 0.027 PDQ-39 *p* < 0.001 At 16 weeks there is a follow-up that shows less significant results.
Carpinella et al. (2017) [35]	2–4	Participants continued their usual medical treatment, which remained stable for the full study period. Assessment and treatment were performed always in “on” medication phase	IG: *n* = 17 Wearable Sensor-Based Biofeedback training. CG: *n* = 20 CPT (Keus et al. (2007) [37] evidence-based recommendations)	3 times/week	45 min	20 sessions 7 weeks	Balance and ability to walk.	BBS, 10MWT. Stabilometric instruments, telequestionnaire of satisfaction in health care.	Statistically significant differences can be seen comparing both groups in favor of IG1. BBS *p* = 0.047 Post-training stabilometric indices *p* = 0.003

IG: Intervention Group; CG Control Group; UPDRS: Unified Parkinson’s Disease Rating Scale; PDQ-39: Parkinson’s Disease Questionnaire; LAPAQ: LASA Physical Activity Questionnaire; 10MWT: 10 Minutes Walking Test; 6MWT: 6 Minutes Walking Test; BBS: Berg Balance Scale; FOG: Freezing Of Gait; TUG: Timed Up and Go; MPAS: Modified Parkinson’s Activity Scale; ADL: Activities of Daily Living; ABC: Activities-specific Balance Confidence Scale; FES: Falls Efficacy Scale; MDS-UPDRS: Movement Disorder Society (MDS)-sponsored revision of the Unified Parkinson’s Disease Rating Scale; FIM: Functional Independence Measure; GFQ: Gait and Falls Questionnaire; SPPB: Short Physical Performance Battery; FAB: Frontal Assessment Battery at the bedside; MRT: Mental Rotation Task; AS: Apathy Scale; SDS: Self-rating Depression Scale; MiniBESTest: Mini-Balance Evaluation Systems Test; MoCa: Montreal Cognitive Assessment; PNF: Proprioceptive Neuromuscular Facilitation; KNGF: Koninklijk Nederlands Genootschap voor Fysiotherapie. * Parkinson’s disease stage was evaluated by the Hoehn & Yahr scale. ** Comparison intervention details were added when they were available in the manuscript.

**Table 4:** Classification of the RCTs according to the measuring instrument, type of intervention and outcome.

Group/Instrument	Studies	Type of Intervention	Outcome
UPDRS-III	Volpe et al. (2014) [30] Volpe et al. (2017) [34]	Aquatic physical therapy	Motor function
	Volpe et al. (2013) [29] Hashimoto et al. (2015) [32]	Dance-based therapy	
TUG (s)	Volpe et al. (2014) [30] Volpe et al. (2017) [34]	Aquatic physical therapy	Balance
	Hashimoto et al. (2015) [32] Rios Romenets et al. (2015) [33]	Dance-based therapy	
BBS	Volpe et al. (2014) [30] Volpe et al. (2017) [34]	Aquatic physical therapy	Balance
	Volpe et al. (2013) [29] Hashimoto et al. (2015) [32]	Dance-based therapy	
ABC	Volpe et al. (2014) [30] Volpe et al. (2017) [34]	Aquatic physical therapy	Balance confidence
FES	Volpe et al. (2014) [30] Volpe et al. (2017) [34]	Aquatic physical therapy	Fall-related self-efficacy
PDQ-39	Volpe et al. (2014) [30] Volpe et al. (2017) [34]	Aquatic physical therapy	Quality of life

UPDRS: Unified Parkinson’s Disease Rating Scale; TUG: Timed Up and Go; BBS: Berg Balance Scale; ABC: Activities-specific Balance Confidence; FES: Falls Efficacy Scale; PDQ: Parkinson’s Disease Questionnaire.

## References

[1] Park J.-H., Kim D.-H., Kwon D.-Y., Choi M., Kim S., Jung J.-H., Han K., Park Y.-G. (2019). Trends in the incidence and prevalence of Parkinson’s disease in Korea: a nationwide, population-based study. BMC Geriatr..

[2] Poewe W., Seppi K., Tanner C.M., Halliday G.M., Brundin P., Volkmann J., Schrag A.-E., Lang A.E. (2017). Parkinson disease. Nat. Rev. Dis. Primers.

[3] Levi V., Carrabba G., Rampini P., Locatelli M. (2015). Short term surgical complications after subthalamic deep brain stimulation for Parkinson’s disease: does old age matter?. BMC Geriatr..

[4] Martínez-Fernández R., Gasca-Salas C.C., Sánchez-Ferro A., Obeso J.A. (2016). Parkinson’s disease: A review. Rev. Médica Clínica Las Condes.

[5] Palazón García R., Gómez del Monte C., Cantero Garlito P.A., Cabañas Elías J., Berrocal Sánchez I. (2001). Treatment protocol in Parkinson’s disease. Rehabilitación.

[6] Miranda C.M., Hudson A.L. (2017). Severe leg edema associated with the use of dopaminergic drugs in Parkinson’s disease. Report of one case. Rev. Med. Chil..

[7] Toosizadeh N., Lei H., Schwenk M., Sherman S.J., Sternberg E., Mohler J., Najafi B. (2015). Does integrative medicine enhance balance in aging adults? Proof of concept for the benefit of electroacupuncture therapy in Parkinson’s disease. Gerontology.

[8] Santos S.M., da Silva R.A., Terra M.B., Almeida I.A., de Melo L.B., Ferraz H.B. (2017). Balance versus resistance training on postural control in patients with Parkinson’s disease: a randomized controlled trial. Eur. J. Phys. Rehabil. Med..

[9] Ganesan M., Sathyaprabha T.N., Gupta A., Pal P.K. (2014). Effect of partial weight-supported treadmill gait training on balance in patients with Parkinson disease. PM&R.

[10] Ricciardi L., Ricciardi D., Lena F., Plotnik M., Petracca M., Barricella S., Bentivoglio A.R., Modugno N., Bernabei R., Fasano A. (2015). Working on asymmetry in Parkinson’s disease: randomized, controlled pilot study. Neurol. Sci..

[11] Picelli A., Melotti C., Origano F., Neri R., Waldner A., Smania N. (2013). Robot-assisted gait training versus equal intensity treadmill training in patients with mild to moderate Parkinson’s disease: A randomized controlled trial. Parkinsonism Relat. Disord..

[12] Liao Y.-Y., Yang Y.-R., Cheng S.-J., Wu Y.-R., Fuh J.-L., Wang R.-Y. (2015). Virtual Reality-Based Training to Improve Obstacle-Crossing Performance and Dynamic Balance in Patients With Parkinson’s Disease. Neurorehabil. Neural Repair.

[13] Hely M.A., Reid W.G.J., Adena M.A., Halliday G.M., Morris J.G.L. (2008). The Sydney Multicenter Study of Parkinson’s disease: The inevitability of dementia at 20 years. Mov. Disord..

[14] Keus S.H.J., Munneke M., Graziano M., Paltamaa J., Pelosin E., Domingos J., Brühlmann S., Ramaswamy B., Prins J., Struiksma C. (2013). European guidelines for physiotherapy in Parkinson’s disease. Mov. Disord..

[15] Hoehn M.M., Yahr M.D. (1967). Parkinsonism: onset, progression, and mortality. Neurology.

[16] Metman L.V., Myre B., Verwey N., Hassin-Baer S., Arzbaecher J., Sierens D., Bakay R. (2004). Test–Retest Reliability of UPDRS-III, Dyskinesia Scales, and Timed Motor Tests in Patients With Advanced Parkinson’ s Disease: An Argument Against Multiple Baseline Assessments. Mov. Disord..

[17] Choi Y.I., Song C.S., Chun B.Y. (2017). Activities of daily living and manual hand dexterity in persons with idiopathic Parkinson disease. J. Phys. Ther. Sci..

[18] Crizzle A.M., Newhouse I.J. (2006). Is Physical Exercise Beneficial for Persons with Parkinson’s Disease?. Clin. J. Sport Med..

[19] Kwakkel G., de Goede C.J.T., van Wegen E.E.H. (2007). Impact of physical therapy for Parkinson’s disease: A critical review of the literature. Parkinsonism Relat. Disord..

[20] Boonstra T.A., van der Kooij H., Munneke M., Bloem B.R. (2008). Gait disorders and balance disturbances in Parkinsonʼs disease: clinical update and pathophysiology. Curr. Opin. Neurol..

[21] Tomlinson C.L., Patel S., Meek C., Herd C.P., Clarke C.E., Stowe R., Shah L., Sackley C., Deane K.H.O., Wheatley K. (2012). Physiotherapy intervention in Parkinson’s disease: systematic review and meta-analysis. BMJ.

[22] Bloem B.R., de Vries N.M., Ebersbach G. (2015). Nonpharmacological treatments for patients with Parkinson’s disease. Mov. Disord..

[23] Hutton B., Catalá-López F., Moher D. (2016). The PRISMA statement extension for systematic reviews incorporating network meta-analysis: PRISMA-NMA. Med. Clin..

[24] (2011). Description of Physical Therapy: Policy Statement. World Confederation for Physical Therapy. Appendix 1. https://www.wcpt.org/policy/ps-descriptionPT.

[25] VanSant A.F. (2006). The International Classification of Functioning, Disability and Health. Pediatr. Phys. Ther..

[26] Maher C.G., Sherrington C., Herbert R.D., Moseley A.M., Elkins M. (2003). Reliability of the PEDro Scale for Rating Quality of Randomized Controlled Trials. Phys. Ther..

[27] Moseley A.M., Herbert R.D., Sherrington C., Maher C.G. (2002). Evidence for physiotherapy practice: A survey of the Physiotherapy Evidence Database (PEDro). Aust. J. Physiother..

[28] van Nimwegen M., Speelman A.D., Overeem S., van de Warrenburg B.P., Smulders K., Dontje M.L., Borm G.F., Backx F.J.G., Bloem B.R., Munneke M. (2013). Promotion of physical activity and fitness in sedentary patients with Parkinson’s disease: randomised controlled trial. BMJ.

[29] Volpe D., Signorini M., Marchetto A., Lynch T., Morris M.E. (2013). A comparison of Irish set dancing and exercises for people with Parkinson’s disease: A phase II feasibility study. BMC Geriatr..

[30] Volpe D., Giantin M.G., Maestri R., Frazzitta G. (2014). Comparing the effects of hydrotherapy and land-based therapy on balance in patients with Parkinson’s disease: a randomized controlled pilot study. Clin. Rehabil..

[31] Monticone M., Ambrosini E., Laurini A., Rocca B., Foti C. (2015). In-patient multidisciplinary rehabilitation for Parkinson’s disease: A randomized controlled trial. Mov. Disord..

[32] Hashimoto H., Takabatake S., Miyaguchi H., Nakanishi H., Naitou Y. (2015). Effects of dance on motor functions, cognitive functions, and mental symptoms of Parkinson’s disease: A quasi-randomized pilot trial. Complement. Ther. Med..

[33] Romenets S.R., Anang J., Fereshtehnejad S.-M., Pelletier A., Postuma R. (2015). Tango for treatment of motor and non-motor manifestations in Parkinson’s disease: A randomized control study. Complement. Ther. Med..

[34] Volpe D., Giantin M.G., Manuela P., Filippetto C., Pelosin E., Abbruzzese G., Antonini A. (2017). Water-based vs. non-water-based physiotherapy for rehabilitation of postural deformities in Parkinson’s disease: A randomized controlled pilot study. Clin. Rehabil..

[35] Carpinella I., Cattaneo D., Bonora G., Bowman T., Martina L., Montesano A., Ferrarin M. (2017). Wearable Sensor-Based Biofeedback Training for Balance and Gait in Parkinson Disease: A Pilot Randomized Controlled Trial. Arch. Phys. Med. Rehabil..

[36] Shujaat F., Soomro N., Khan M. (2014). The effectiveness of Kayaking exercises as compared to general mobility exercises in reducing axial rigidity and improve bed mobility in early to mid stage of Parkinson’s disease. Pak. J. Med. Sci..

[37] Keus S.H.J., Bloem B.R., Hendriks E.J.M., Bredero-Cohen A.B., Munneke M. (2007). Evidence-based analysis of physical therapy in Parkinson’s disease with recommendations for practice and research. Mov. Disord..

[38] Podsiadlo D., Richardson S. (1991). The Timed ”Up & Go”: A test of basic functional mobility for frail elderly persons. J. Am. Geriatr. Soc..

[39] Blum L., Korner-Bitensky N. (2008). Usefulness of the Berg Balance Scale in Stroke Rehabilitation: A Systematic Review. Phys. Ther..

[40] Shanahan J., Morris M.E., Bhriain O.N., Volpe D., Lynch T., Clifford A.M. (2017). Dancing for Parkinson Disease: A Randomized Trial of Irish Set Dancing Compared With Usual Care. Arch. Phys. Med. Rehabil..

[41] Aguiar L.P.C., da Rocha P.A., Morris M. (2016). Therapeutic Dancing for Parkinson’s Disease. Int. J. Gerontol..

[42] Jonasson S.B., Nilsson M.H., Lexell J. (2014). Psychometric properties of four fear of falling rating scales in people with Parkinson’s disease. BMC Geriatr..

[43] Methajarunon P., Eitivipart C., Diver C.J., Foongchomcheay A. (2016). Systematic review of published studies on aquatic exercise for balance in patients with multiple sclerosis, Parkinson’s disease, and hemiplegia. Hong Kong Physiother. J..

[44] Kemper K.J. (2019). What’s new in complementary therapies for Parkinson’s disease?. Complement Ther. Med..

[45] Zhang Q., Hu J., Wei L., Jia Y., Jin Y. (2019). Effects of dance therapy on cognitive and mood symptoms in people with Parkinson’s disease: A systematic review and meta-analysis. Complement. Ther. Clin. Pract..

[46] dos Santos Delabary M., Komeroski I.G., Monteiro E.P., Costa R.R., Haas A.N. (2018). Effects of dance practice on functional mobility, motor symptoms and quality of life in people with Parkinson’s disease: a systematic review with meta-analysis. Aging Clin. Exp. Res..

